# Does serum interleukin-6 guide the diagnosis of persistent infection in two-stage hip revision for periprosthetic joint infection?

**DOI:** 10.1186/s13018-019-1448-7

**Published:** 2019-11-11

**Authors:** Peng-Fei Qu, Chi Xu, Jun Fu, Rui Li, Wei Chai, Ji-Ying Chen

**Affiliations:** Department of Orthopedics, First Medical Center, General Hospital of People’s Liberation Army, No.28 Fuxing Road, Haidian District, Beijing, 100853 China

**Keywords:** Periprosthetic joint infection, Two-stage revision arthroplasty, Diagnosis, Interleukin-6

## Abstract

**Purpose:**

The diagnosis of persistent infection at reimplantation of two-stage revision arthroplasty for periprosthetic joint infection (PJI) remains challenging. Several studies have shown the benefit of serum interleukin-6 (IL-6) in diagnosing periprosthetic joint infection (PJI). Recent data indicated serum IL-6 could be promising in differentiating persistent infection. The purpose of this study was to validate the efficacy of serum IL-6 in diagnosing persistent infection at reimplantation.

**Methods:**

A retrospective review of 86 PJI patients with a two-stage exchanged hip arthroplasty from 2013 to 2017 was conducted. Persistent infection was defined using the modified Musculoskeletal Infection Society (MSIS) criteria combined with follow-up results. Serum IL-6 at reimplantation were collected and compared among patients with or without persistent infection. Receiver operating characteristic (ROC) curves were generated to evaluate the diagnostic performance and optimal cut-off value of serum IL-6 at reimplantation.

**Results:**

Sixteen cases were diagnosed as persistent infection at reimplantation. There was no significant difference in serum IL-6 levels between cases with persistent infection and controls (7.89 pg/ml vs. 5.56 pg/ml; *P* = 0.179). The area under the ROC curve (AUC) for serum IL-6 in diagnosing persistent infection at reimplantation was 0.59 (95% confidential interval [CI] 0.40–0.77). With the calculated threshold set at 8.12 pg/ml, the corresponding sensitivity, specificity, positive predictive value, and negative predictive values were 38%, 88%, 38%, and 87%, respectively.

**Conclusion:**

Serum IL-6 is inadequate in diagnosing persistent infection at reimplantation for two-stage revision arthroplasty. With the serum IL-6 threshold set at 8.12 pg/ml, the specificity to rule out persistent infection is high, but the sensitivity to predict persistent infection is not satisfactory.

## Introduction

Periprosthetic joint infection (PJI) remains a disastrous complication following total joint arthroplasty (TJA) with the incidence of 1.2–4.6% [1, 2]. The management of PJI is challenging. Two-stage revision arthroplasty, involving resection of implants with an antibiotic-loaded spacer insertion followed by reimplantation of a new prosthesis, remains the “golden standard” for chronic PJI [3]. However, studies with large sample sizes frequently reported the failure rate of two-stage revision arthroplasty were more than 20% [4].

It is critical to determine the optimal timing of reimplantation in two-stage revision arthroplasty [5, 6], which guides whether to implant a new prosthesis or spacer exchange. However, as far as we know, biomarkers used for diagnosis of PJI, e.g., serum erythrocyte sedimentation rate (ESR), C-reactive protein (CRP), synovial fluid analysis, and intraoperative frozen section, have limited values in predicting persistent infection at reimplantation [7–10]. Furthermore, a lack of accessible synovial fluid or a “dry tap” is not rare in patients with an antibiotic-loaded spacer in situ [11], which can make the decision-making process of reimplantation more complicated.

Studies have shown the promising efficacy of serum IL-6 in diagnosing PJI after primary arthroplasty [12, 13]. However, the value of serum IL-6 in diagnosing persistent infection remains unclear. Recently, a prospective study by Hoell et al. evaluated the diagnostic performance of serum interleukin-6 (IL-6) in identifying persistent infection at reimplantation. They indicated the positive predictive value of IL-6 was 90.9% with the threshold set at 13 pg/ml, and the negative predictive value of 0.921 with the threshold set at 8 pg/ml. Thus, they indicated serum IL-6 had a good efficacy in diagnosing persistent infection before second-stage of revision [14]. However, no further study has been conducted to validate these findings. Therefore, we performed a retrospective study to identify the value of serum IL-6 in diagnosing persistent infection at the timing of reimplantation.

## Methods

### Patient demography and characteristics

We retrospectively reviewed 99 patients (99 joints) with chronic PJI following primary total hip arthroplasty who underwent a two-stage revision arthroplasty from 2013 to 2017. The diagnosis of PJI was based on the Musculoskeletal Infection Society (MSIS) for PJI [15]. The minimum follow-up was 1 year. Patients with the history of malignance or immunosuppressive disease, and those without serum IL-6 at reimplantation were excluded. After the aforementioned exclusion, 86 patients were included in the final analysis, which was grouped into two groups: 16 patients in infection persistent group and 70 patients in infection eradicated group. Patient’s age, gender, and BMI values were compared, and there was no significant difference between the two groups (Table 1).

**Table 1:** Comparison of patient characteristics between infection persistent group and infection eradicated group

	Infection persistent group (*n* = 16)	Infection eradicated group (*n* = 70)	*P* value
Age (year)	56 (28–72)	54 (18–86)	0.67 (*T* test)
BMI (kg/m^2^)	26 (19.7–32.4)	24 (15.8–32.9)	0.15 (*T* test)
Gender			0.60 (chi-square)
Male	8	40	
Female	8	30	

For most patients in the infection persistent group, organisms were found at the timing of first-stage surgery and at the timing of reimplantation. Organisms found at the timing of reimplantation may vary from the first stage. Some patients were diagnosed with persistent infection by the presence of sinus or infection recurrence, but with negative culture, the negative results are recognized as false negative (Table 2).

**Table 2:** Organisms found in the first- and second-stage surgery in persistent infection patients

First-stage culture-positive bacteria	Second-stage culture-positive bacteria
*Staphylococcus aureus*	*Staphylococcus aureus*
*Staphylococcus aureus*	*Staphylococcus aureus*
*Staphylococcus aureus*	None
*Staphylococcus aureus*, *Enterococcus faecalis*	*Moraxella osloensis*
*Staphylococcus epidermidis*	*Staphylococcus hominis*
*Staphylococcus epidermidis*	*Micrococcus luteus*/L
*Peptoniphilus asaccharolyticus, Staphylococcus epidermidis*	Nocardia
*Streptococcus viridans*	*Staphylococcus epidermidis*
*Staphylococcus lugdunensis*	None
None	*Staphylococcus warneri*
None	*Staphylococcus haemolyticus*
None	*Staphylococcus epidermidis, Staphylococcus aurantia*
None	*Staphylococcus hominis*
None	None
None	None
None	None

### Tests and materials

Serum IL-6 was routinely obtained before reimplantation in our institution by using a uniform instrument and reagents (Immulite 2000, Siemens Medical Solutions Diagnostics GmbH, Eschborn, Germany). For patients with repeat IL-6 tests, the latest value before reimplantation was collected. Other medical records were reviewed manually, including age, gender, body mass index (BMI), serum CRP and ESR values, intraoperative cultures, and histologic results.

### Treatment protocol

Patients were treated with standard two-stage revision arthroplasty by three experienced surgeons in our institution, which included removal of prosthesis, thorough debridement of infected tissues, insertion of an antibiotic-loaded cement spacer (10% vancomycin, 5% meropenem), administration of microbe-sensitive antibiotics (intravenous 6 weeks plus oral 6 weeks), and the final reimplantation. After that, all patients received 2 to 6 weeks of sensitive antibiotic treatment according to the intraoperative culture result of reimplantation.

### Definition of persistent infection

**Fig. 1:**
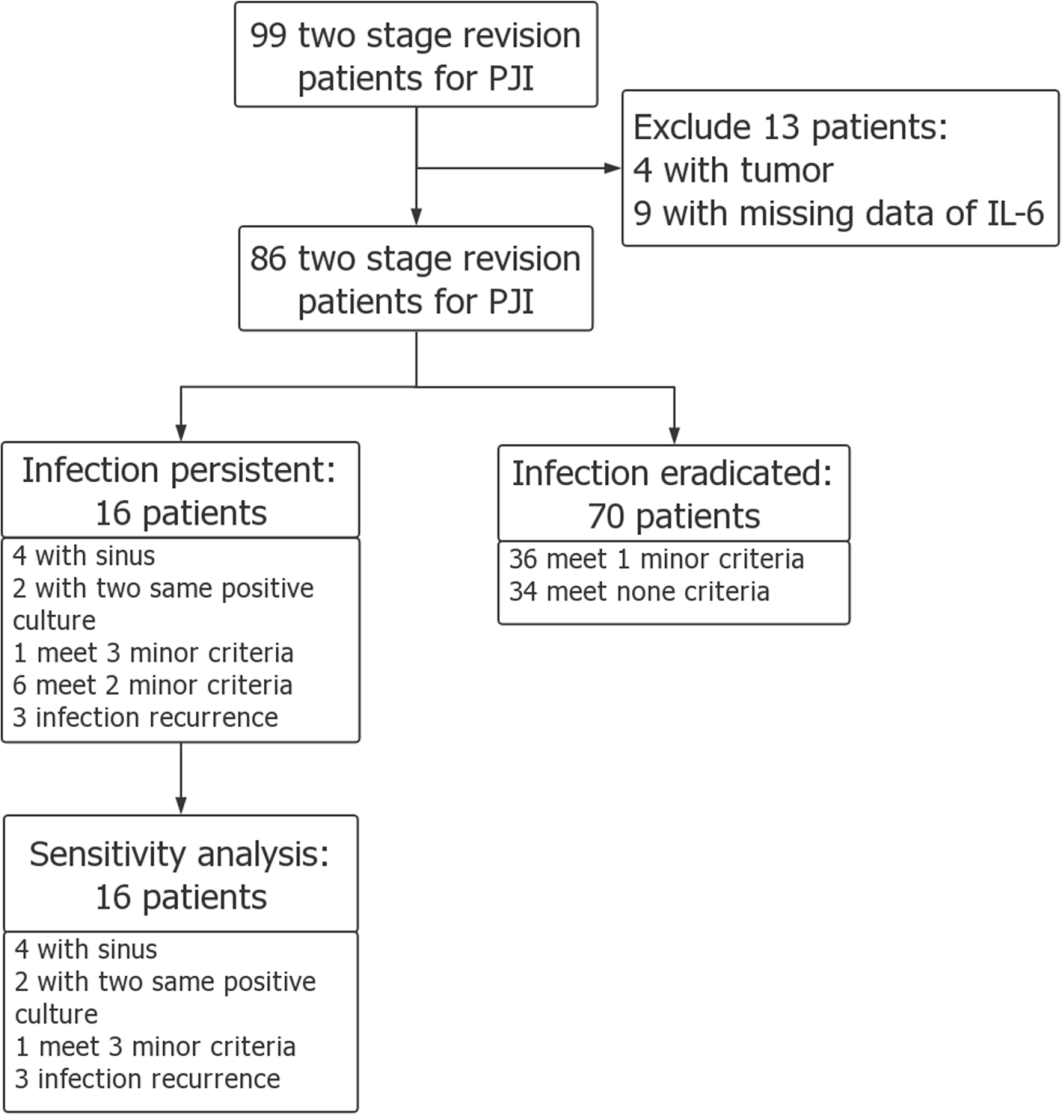
Flowchart for patients’ group

MSIS criteria are the most commonly used criteria to diagnose PJI after primary arthroplasty. It is also helpful in diagnosing persistent infection. Some studies have used modified MSIS criteria to define persistent infection [7, 10, 11, 16]. We combined modified MSIS criteria with follow-up results to define persistent infection, which is supposed to have a higher sensitivity and specificity. Persistent infection diagnosis should meet the major criteria: presence of sinus connecting to the outside or two intraoperative culture-positive results with the same bacteria or met at least two of the following three minor criteria: (1) preoperative serum ESR > 30 mm/h and CRP > 10 mg/L, (2) one positive intraoperative culture, and (3) average > 5 neutrophils on at least 5 high power field view of frozen pathology section. Patients who have encountered infection recurrence after revision were also defined as persistent infection. Infection eradication was defined as met less than two minor criteria (Fig. 1).

### Statistical analysis

Statistical analysis was performed using IBM-SPSS Statistics v. 23 (IBM, Armonk, New York). Continuous variables like age and BMI values were compared using *T* test. A categorical variable like gender was compared using chi-square test. ROC curve was plotted to assess the diagnostic performance of serum IL-6. The optimal cut-off value was set to calculate corresponding sensitivity, specificity, positive predictive value (PPV), and negative predictive value (NPV).

### Sensitivity analysis

For no gold standard to diagnose persistent infection, a sensitivity analysis was done to verify the results. We reevaluate the diagnostic performance of serum IL-6 by a stricter definition. Under this condition, persistent infection diagnosis should meet the major criteria: the presence of sinus connecting to the outside or two culture-positive results with the same bacteria or met at all three minor criteria: (1) preoperative serum ESR > 30 mm/h and CRP > 10 mg/L, (2) one positive intraoperative culture, and (3) average > 5 neutrophils on at least 5 high power field view of frozen pathology section. Patients who have encountered infection recurrence after revision were also defined as persistent infection. Then, the corresponding ROC curve was plotted and statistical analysis was performed.

## Results

**Fig. 2:**
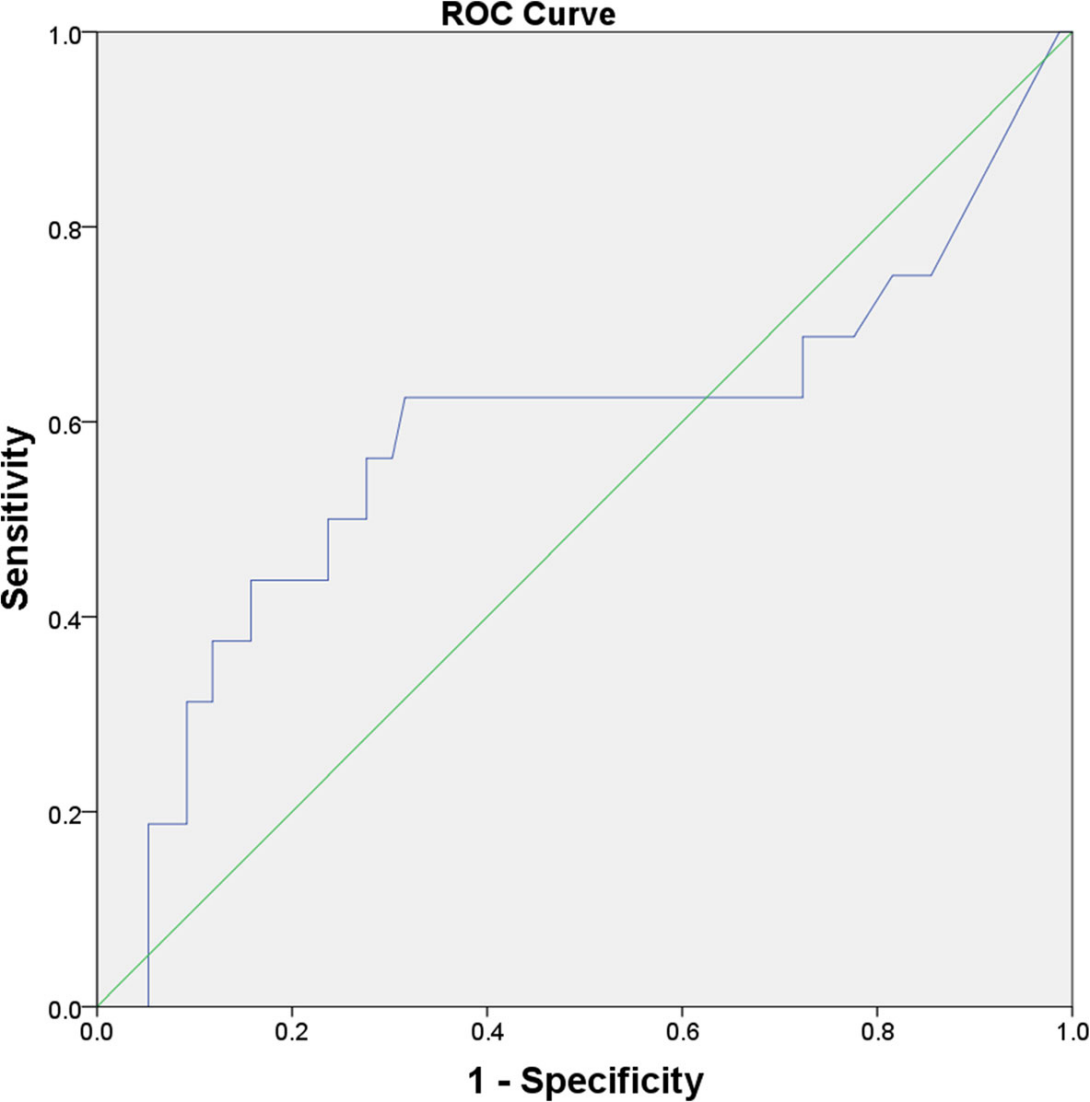
ROC curve for serum IL-6

There was no significant difference in serum IL-6 values between cases with persistent infection and the controls (7.89 pg/ml vs. 5.56 pg/ml; *P* = 0.179). ROC curve was plotted to calculate the diagnostic performance of serum IL-6, and the AUC for serum IL-6 is 0.59 (95% CI 0.40–0.77) (Fig. 2). We found the optimal cut-off value for serum IL-6 at 8.12 pg/ml, and the corresponding sensitivity, specificity, PPV, and NPV were 38%, 88%, 38%, and 87%, respectively. Since ESR, serum CRP, and intraoperative frozen pathology were included in the modified MSIS diagnostic criteria, they were not taken into the statistical analysis.

### Sensitivity analysis

**Fig. 3:**
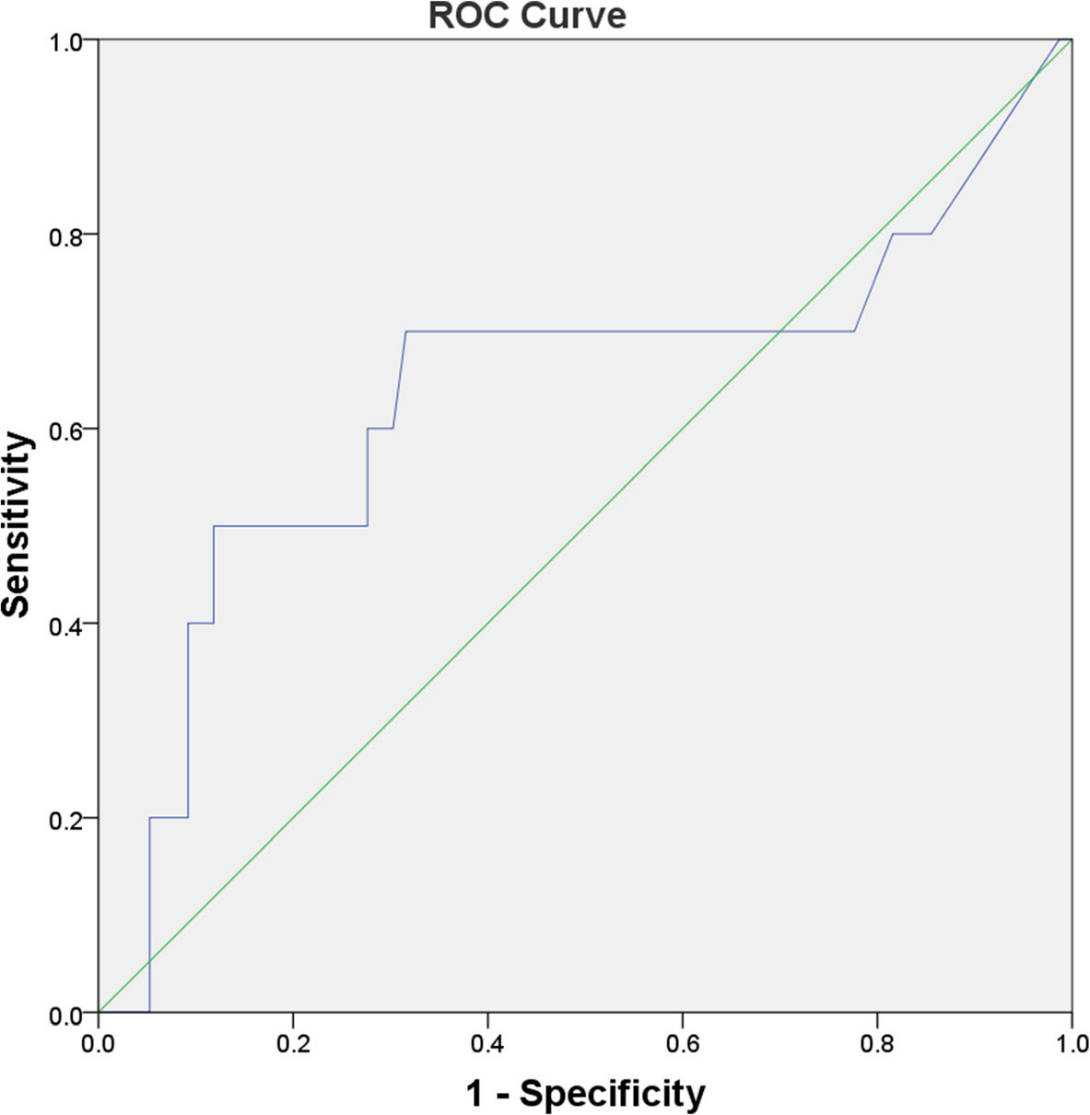
ROC curve for serum IL-6 in sensitivity analysis

With the stricter definition of persistent infection, 11.6% (10/86) patients are defined as persistent infection. There was no significant difference in serum IL-6 values between cases with persistent infection and the controls (9.03 pg/ml vs. 5.56 pg/ml; *P* = 0.172). ROC curve was plotted to verify the diagnostic efficacy of serum IL-6 for persistent infection, and the AUC for serum IL-6 is 0.64 (95% CI 0.41–0.86) (Fig. 3). We found the same best cut-off value for serum IL-6 at 8.12 pg/ml, and the corresponding sensitivity, specificity, PPV, and NPV were 50%, 88%, 50%, and 93%, respectively.

### Outcomes

**Fig. 4:**
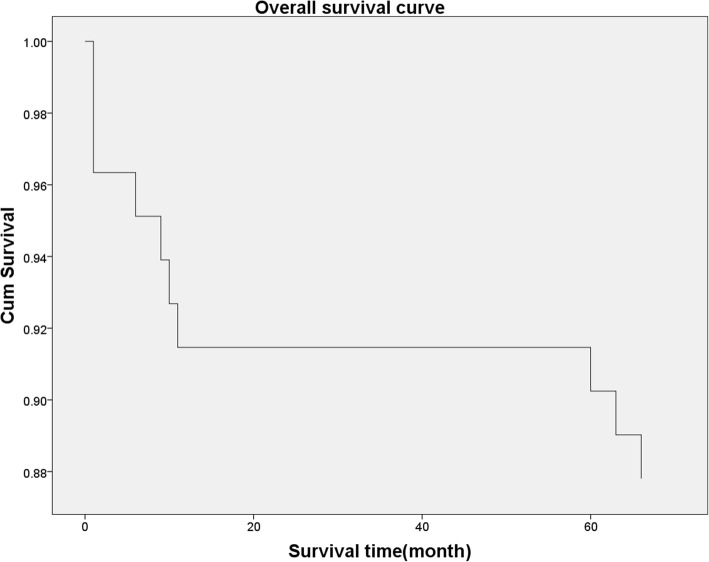
Overall survival curve for two-stage revision patients

Eighty-two in 86 patients (95.3%) were followed with a mean follow-up time of 4 years. According to the Delphi criteria, treatment success for PJI was defined as: (1) infection eradication, (2) no subsequent surgical intervention for infection, and (3) no occurrence of PJI-related mortality [17]. Three cases failed because of infection recurrence, 2 cases underwent two-stage revision, and 1 case received debridement, antibiotics, and implant retention. The infection eradication rate for two-stage revision arthroplasty in our institution is 96.3%. Seven cases failed because of other reasons and underwent revision (1 for the abnormal sound of the prosthesis, 3 for aseptic loosening, 3 for hip dislocation). The overall success rate for two-stage revision arthroplasty in our institution is 87.8%. Overall survival curve was plotted to show detailed information (Fig. 4).

## Discussion

Persistent infection before reimplantation is an important indicator of poor prognosis of two-stage revision. Serum CRP, ESR, and alpha-defensin have shown diagnostic abilities for PJI after primary arthroplasty, but have limited efficacy in diagnosing persistent infection at reimplantation [9, 18–20]. After reviewing reported parameters for preoperative diagnosis, we found that serum IL-6 is a promising maker [21, 22]. In this retrospective study, we have found that serum IL-6 has a high specificity and NPV with the cut-off value set at 8.12 pg/ml. When serum IL-6 < 8.12 pg/ml, there is a high probability that the infection has been cleared. What is more, there are only 2 cases in our study with serum IL-6 value between the gray area of 9 pg/ml to 12 pg/ml. These results are consistent with previous findings [14].

However, we did not find a cut-off value of 13 pg/mL with high sensitivity and PPV, which is different from Hoell’s study.

To further validate the diagnostic performance of serum IL-6, we conducted a sensitivity analysis and found similar results. With the same cut-off value for serum IL-6, the specificity was high and the sensitivity had elevated slightly to 50% but was still not enough.

These results illustrated the limited efficacy of serum IL-6 in the diagnosis of persistent infection before reimplantation of two-stage revision, which may be owing to the long-term antibiotic use and low-toxic PJI.

The difference may also be explained by the different definitions of persistent infection. Hoell’s study defined persistent infection by at least two positive cultures with an antibiotic spacer in place, from at least three tissue samples obtained during the second-stage procedure. However, because of the long-term use of antibiotics in the revision interval, the culture may be false negative. For those patients, using modified MSIS criteria combined with follow-up results may improve the accuracy of the definition criteria.

There are some limitations in our study. Firstly, the modified MSIS criteria did not include data of synovial fluid white blood cell (WBC) count and synovial fluid polymorphonuclear neutrophil percentage (PMN%), which is a suboptimal choice. So, it needed an additional sensitivity analysis. Secondly, there were limited cases in our study and further studies are needed to explain the controversial results. In addition, 4 patients (4.65%) were not available for follow-up; thus, some infection recurrence may not be recorded and the overall rate of infection recurrence may be affected.

## Conclusion

This study shows that the value of serum IL-6 has limited efficacy in differentiating persistent infection before reimplantation of two-stage revision. With existing parameters, we still cannot predict persistent infection precisely and determine the optimal timing for reimplantation. However, further studies are needed to validate our results.

## Data Availability

We do not wish to share our data, because some of the patient’s data regarding individual privacy, and according to the policy of our hospital, the data could not be shared with others without permission.

## Abbreviations

AUC: Areas under the curves
CRP: C-reactive protein
ESR: Erythrocyte sedimentation rate
IL-6: Interleukin-6
MSIS: Musculoskeletal Infection Society
NPV: Negative predictive value
PJI: Periprosthetic joint infection
PPV: Positive predictive value
ROC: Receiver operating characteristic curves
